# The Æsthetic Correction of Facial Contours in the Practice of Dental Orthopedia

**Published:** 1895-12

**Authors:** 


### The Æsthetic Correction of Facial Contours in the Practice of Dental Orthopedia.

(*Continued from Page* 561.)

### DISCUSSION.

George H. Cushing, Chicago: I am not aware that there can be much discussion upon a paper of this character. I do not know that there are any technical objections to the position that the paper assumes, as to the possibility of moving the teeth *en phalanx* bodily, the sockets as well as the teeth. If there are any such objections, they must fall before the positive evidence of clinical observation.

I think the paper shows conclusively that, as Dr. Farrar remarked, "this demonstrates an era of advance in orthopœdic surgery." I think we are most indebted to Dr. Case for an intelligent study of the mechanical principles which govern the movements of the teeth by applied force, in connection with the fact which he has demonstrated, of the possibility of moving the teeth and the processes together. You have seen what he has accomplished, and these models and drawings speak more eloquently than any language can express.

Two of these cases I have seen under treatment from the first. I cannot begin to tell you the extent of the improvement in the facial expression of No. 4. The maxillary bone and the process were so receded that there were depressions each side of the median line so deep that you could lay your finger in them. Those are now very nearly two-thirds obliterated, I should think, and though this mask shows a wonderful improvement, it does not show fully the great change which has been effected, although, as he told you, that was one of the cases so difficult to manage because of the rapid absorption of the process from the pressure of the roots. I think he hopes in time to entirely obliterate the deep depressions under the alæ of the nose. From my observation, so far as the case has progressed, I have no doubt that he will succeed.

Of the other case, No. 9, I may say that these casts especially do not begin to show the improvement that has taken place in the short time in which the patient has been under treatment. The boy presented a very disagreeable aspect, as you see here. There is one feature of the case which the author of the paper did not refer to; it is a feature which is very striking. The boy had the habit of dropping his mouth open continually. He does not do this at all now. I do not know why the movement of these teeth and the contouring of the face by this application of force should have produced that change, but it is a fact that it has. The boy now keeps his mouth closed as other people do. With his chin apparently protruding, owing to the lack of development of the superior maxillæ, and the mouth open all the while, you may imagine how very unpleasantly he must have presented himself to his friends. He is now a pretty respectable looking boy, and he was very far from that when he first went into Dr. Case's hands. His family are exceedingly delighted, as they may well be.

Dr. James G. Reid, Chicago: I suppose a few of you will go home imbued with the idea of being able to accomplish what has been shown here to-night by Dr. Case—who is master of the situation; attained only by years of experience and a close study of the physical forces employed and their application, how to direct those forces to the moving of teeth bodily in their sockets. Dr. Case will sit down and figure out on paper, by geometrical and mathematical processes, the direction and magnitude of force at the different points of a regulating appliance and the probable influence it will exert. When you understand that, you will appreciate the success of which he has shown you evidence by these models. It looks easy, when you hear him explain it, but if you attempt it you will be deceived, if you suppose it to be easy. I have seen many of these cases from time to time every few days. Possibly you may feel inclined to doubt some of the results, but I say, as Dr. Cushing has said, that these models do not begin to show you the improvement, and in order to appreciate it you should see the individuals themselves.

Dr. Louis Ottofy, Chicago: The first thing which arrested my attention in listening to the excellent paper of Dr. Case was the introduction of the word Orthopedia into our dental nomenclature. I presume that when Dr. Case commenced the practice of Orthodontia, he did it on the same lines in which it was generally practiced; that is, the only aim of the operator was to straighten the teeth, principally the anterior teeth, so that when the mouth of the patient is open, they appear regular.

As Orthodontia advanced, an effort was made to also make the teeth more useful for the purposes of mastication, and attention was directed to the attainment of that result as much as to improving the appearance. That is the point to which it seems to have been carried, until Dr. Case took up the subject. He has certainly carried it far beyond that, and the term he has applied to it seems to me to be one that is very well adapted for the expression of what he really accomplishes. Aside from what has been referred to to-night, more is realized than any of us comprehend; and that is, in giving character to comparatively characterless faces. Take, for instance, the case of the boy who has been mentioned. A man with any noticeable defect must battle against more odds than the man who is without defects. Because the face looks characterless is not necessarily an indication that the individual lacks character, or that he may not develop more than ordinary ability. The fact that a man is crippled does not detract from his mental ability. He may be just as able as the man who is not crippled; but when this idea is applied to the face, it is a more serious question. We judge much by the countenance of the individual. So the addition of the appearance of character to the face is important. In the case of a woman there is also the creation of beauty. I have seen a number of the cases used for illustration by Dr. Case, and I am satisfied that the casts which we have before us do not give an adequate idea of the improved appearance. When we look at the models of a set of teeth, showing the condition before and after treatment, we are able to appreciate the change, if the model is correct; but in taking the impression of a face a different factor is involved. Take, for instance, Case 1. Just as soon as the mind of that individual becomes active and the muscles of the face play, every deformed and defective feature is more strongly brought to the attention of the beholder. The individual appears homely or repulsive. This is generally true of deformity, but not necessarily of *homely, regular* features. When the cast is taken, that homeliness is largely obliterated because the face (during the making of the cast) must of necessity be in repose. On the other hand, after the face has been beautified you have in the cast a cold, emotionless face, regular in appearance, but lacking the beauty which mirrors the emotions of the mind as the face is in motion.

The apparatus which Dr. Case employs seems complicated, but, like all complicated things, is simple when understood.

However, that matters little. The point which I wish specially to mention is, that the patients wear these appliances without any apparent annoyance, with far more comfort than appliances made earlier and which were supposed to be very simple.

I would suggest to the essayist that, in connection with the preparation of his models and casts of faces, he also take actual measurements both of the expansion and retraction of the jaws, and of the faces themselves, so that we may have for future reference some absolutely tangible and indestructible evidence of what progress has been made in this line.

Dr. W. H. Jackson, Ann Arbor, Mich: As to the moving forward of the process, I know it can be done as the doctor has said. I have in my own practice produced the same results, not in exactly the same way, but effectually, even where the intermaxillary process developed out of place, so that it left both cuspids standing out prominently and the incisors setting back even with the first bicuspids. The whole process, with those four teeth, was moved forward as a body, so that they came out even with the cuspids. There was a depression of the upper lip. The patient had been advised to have the lower bicuspids extracted and the lower teeth drawn back; but I told her that would still leave the expressionless upper lip. If you drew the lower lip back even with it, it would leave a still worse condition, with the prominence of the chin and the very great prominence of the nose; I proposed to her that, although it would take a long time to do it, it would be much better for her to have the superior incisors carried forward. It took between ten and eleven months to move them forward, without doing it so fast to cause absorption, as Dr. Case has said. If you move them too rapidly, you may cause absorption in almost any case; and if you apply too great force, you may even cause a separation of the process between the teeth and crowd the front bony plate forward.

A great many times in regulating the teeth we come across cases where they are so rigid that it seems impossible to move them. You will find the rapidity with which you can move teeth depends upon the amount of connective tissue between the tooth and the alveolar process. If you have a considerable portion of connective tissue between the tooth and the alveolar process, it will move faster. If, on the other hand, there is very little, with little blood-supply, it is very difficult to get up irritation enough to cause absorption and the moving of the tooth.

Dr. J. Taft, Cincinnati: I wish to ask Dr. Case if he has not found that there is frequently a change in speech as the result of these changes in contour?

Dr. Case: Yes.

Dr. Taft: And that the speech is always improved and never made worse?

Dr. Case: I think that is true.

Dr. Taft: Sometimes in the regulation of teeth, difficulty will arise because of irritation, or inflammation. I presume, however, after listening to the paper, that the process is not pushed by Dr. Case to the extent of inducing irritation. I should like to ask him, if he has found that there is a disposition in the tissues, and does he not find it necessary sometimes to regulate the movements and the adjustment of the appliances because of that predisposition?

Dr. Case: Yes.

Dr. Taft: Is it ever the case that special diseased conditions arise in the ordinary performance of this work?

Dr. Case: I have never had any experience in that direction.

Dr. Taft: I presume your work has been so well regulated that results of that kind have not occurred. I know that oftentimes in the regulation of teeth, when the pressure is great and the movement rapid, inflammation is set up, and I have known cases of teeth being devitalized, in that way. But I have no doubt that Dr. Case's appreciation of conditions that might arise would be so delicate that every difficulty of that kind would be avoided. A dentist sometimes says "I moved that tooth a distance equal to its whole diameter in eight or ten days." "Did you have any trouble?" "Yes, it was sore, but what of it, it got along." That occurs sometimes by the pressure of the tooth against the socket. But here the whole tooth is moved bodily and in its vertical position, from one position on to another, carrying with it the outer plate of the alveolus, the inner plate of the alveolus either following or the space being filled up—the space that is made by the movement of the tooth. If that process was pushed very rapidly, I can conceive that in some cases there might be irritation. Then again, here are the vessels, the tissues entering the end of the root, that move, and the question occurs may not that sometimes be affected by this movement? You have moved the tooth a quarter of an inch, moved the foramen a line or two from the original position, and all that must be carried along with it, the tissues must be distended to accommodate this movement. I suppose that is the case where they are carried along in that manner.

Dr. Case: Not exactly that. If the entire bone is bent forward with the end of the root, then the point of the root is not carried through the bone, and the foramen that comes down, carrying the vessels to the tooth, is also carried forward, is not left in the original position. That only occurs in those instances where the movement occurs only by absorption of the socket; and whenever that occurs, as I said before, the movement is always very slow; and I do not think I have moved the roots of a tooth—unless in one case—over an eighth of an inch. But where the entire bone can be bent forward, then the movement is always very rapid and quite extensive. The entire vessels and the bone itself seem to be carried along together, and that also is a reason for not expecting so much inflammation as you speak of. And one of the great reasons why inflammation does not occur is, that the method of attaching the appliances and the method of applying the force hold these teeth firmly in their grasp. The force usually applied by those who regulate the teeth is a screw force, which carries the tooth forward. I used to have the same terrible condition of inflammation you speak of when I used the old methods, the pressure and ligatures and rubber bands, but now I do not experience that trouble so often.

Dr. Taft: I can readily see that you have a grasp upon the whole tooth, and it is carried along so that the force is distributed throughout the parts.

Dr. Case: Yes.

Dr. Taft: The whole moves along together.

Dr. Case: Yes.

Dr. Taft: And the vessels, and the tissue attached to the body of the tooth, accommodate themselves to the movement of the tooth.

Now, some cases seem to invite an operation of this kind. How often a bicuspid, or sometimes a first molar, may be removed early in life, and the second molar will move bodily right up to the posterior surface of the second biscuspid. That is the same thing. It moves up from its original position and sets right against the bicuspid. Not always, but often-times there is a successful effort of this sort by nature. And whenever nature makes an effort for repair of this kind, the aim should be to aid it. There will oftentimes, of course, be obstacles in the way that are difficult to overcome; but there often seems to be an inclination on the part of nature to help in these things, and in the case of deformities, a tendency to work to a certain type, to repair a deformity.

The presentation of these cases and methods by Dr. Case has been of great interest to us all, though probably but few of us will ever attempt to do just the things he does, and in the same way; but it presents to us all a striking lesson, and the query will naturally arise in the mind of every practitioner, "Can I not do something to help the afflicted patients?" Dr. Case restores beauty. "Can I not help this deformity a little, by this or that arrangement?" And sogradually, I have no doubt, if this subject is kept before the profession, as Dr. Case is doing by the presentation of these cases at our meeting here, and then throughout the country, the result will be, not only the presentation of what Dr. Case and few others can do, but it will be helpful to us all, by showing what can be done. And then the question will arise, "Can I not also do something to relieve this suffering patient." And so it will be helpful to every earnest, progressive dentist, and to our patients.

Dr. J. G. Templeton, Pittsburg: I should like to hear from Dr. Case in reference to the use of retaining applances, and what he thinks is necessary in this line.

Dr. Case: Retaining the teeth in the position to which you bring them is quite as important as the regulation of them, and is, I have sometimes thought, the most difficult part of the whole operation. I wish I had time to describe some of the new appliances for retaining teeth which I have recently thought out. Patients object very much to wearing appliances that show in the mouth. They want to get rid of the whole thing as soon as possible, and I have invented some new things that hold the teeth perfectly in place and yet do not show any more than a gold filling between the teeth. At some future time I shall fully describe them, so that you will all understand.

Dr. Garrett Newkirk, Chicago: I wish simply to add a word of testimony and of appreciation, concerning the work of Dr. Case. I have paid considerable attention to the regulation of teeth, and my work in that line has been a source of great satisfaction to me and to my patients. I wish to call attention to the principle of the appliance for which, it seems to me, Dr. Case is entitled to the exclusive credit. The advisability of moving the end of the root has been understood for a long time, but just how to construct an appliance which would accomplish that result, and give the operator perfect control of the whole matter of the movement of the teeth, is something which no one has been able to accomplish satisfactorily heretofore. But Dr. Case has certainly solved the problem through a recognition of the mechanical principles involved. So far as I know, no one ever thought before of banding the tooth and having a lever carried from that band up to this point (referring to the model) in order to give him the proper direction of force. He controls it by this traction bar or wire. It is simply a study of the principle of the lever as variously applied. By this traction wire he controls the point of the tooth absolutely. By this nut he controls his force positively. He knows just what he is doing.

Now, a word as to the production of inflammation. With this kind of an appliance the danger is reduced to a minimum, because the tooth is held firmly. There is no action and reaction. There is no uncertainty of movement. On a No. 18 wire there are about ninety threads to the inch, I think, so that you know absolutely, if you give the nut one revolution, that you have a forward motion of one-ninetieth of an inch, while a half-turn gives one one hundred and eightieth of an inch. In almost any case you can control the amount of force, so that there is no possible danger of exciting inflammation. There is no more comparison between this sort of an appliance and our old regulating devices than there is between a nice watch and the old-fashioned threshing-machine. This is scientific, delicate, and positive. You know just what you are doing. So far as I am able to apply mechanical principles, in the few cases that I undertake, I am doing it on the same line with Dr. Case's scheme.

Dr. J. Taft, Cincinnati: One thing which ought to be noted in connection with these appliances is that the movement is positive. The tooth is moved up to a certain point and retained there until the parts accommodate themselves to it, until the tissue impinged upon has accommodated itself to the new condition. It is very different from the spring, or the rubber bands so often used in the correction of irregularities, and there is not anything like the liability or irritation that there is from the spring. It is a positive movement and then a rest, allowing time for accommodation of the parts.

Dr. S. N. Hoff, Ann Arbor, Mich: As I sat here to-night listening to this paper, I wondered if Dr. Case had not fixed these things up and brought them over here to make us believe that he was doing something in the line of art rather than practical dentistry. Now, I believe he is doing both. It looks to me as though he was cultivating art, trying to assist nature to develop among his patients the beauties of art; and it would not be surprising to me, if after a while, to dentistry should be given the credit for a great advance in art, in a way that will take the laurels from the old masters.

As I looked at these drawings and as the discussion was going on, the question came to me, "How is all this beautiful development of the face accomplished?" As I understand from the paper, it is not accomplished by moving the tooth through the alveolar process, but by moving the bones themselves; and if it is accomplished in that way, what bones are moved? You can, not move the bones of the face around without leaving spaces- and the question arises, what fills the spaces, and where is this separation made? It occurred to me that, possibly, the cuspids were moved but little, and that this movement—especially where the process and the contour were developed—came about from the separation of the intermaxillary bone from the maxillary. But how can that be? The intermaxillary bone is united to the maxillary bone at a very early age, before the permanent teeth are developed, and the doctor states that many of these cases are fifteen, sixteen, or seventeen years old, and some older. At that period the intermaxillary and maxillary bones are certainly united, so that there must be a fracture or stretching of this union of the maxillary and the intermaxillary bones between the lateral incisors and cuspids. The casts and models clearly indicate that it is the intermaxillary bones tha are moved forward. I think if thac is the case, if that is what is done, it will explain largely the way in which the force should be applied to develop this contour. The forcing out of the intermaxillary bones bodily would account for the lack of irritation and injurious effects to the blood-vessels and nerves, because we know that the blood and nerve-supply of the cuspids is different from that of the incisors or the bicuspids. It seemed to me that this moving-out process is one comparatively easy to explain, on the theory of fracturing or separating the bones along this original developmental fissure. How can we explain the contrary process—the forcing back of the bone? There could be no fracture of the bones in that way. In that case you must certainly move the teeth. I cannot see that anything could be accomplished in condensing the bones, but that you must certainly in that case move the teeth into the bone, or, by the continued pressure, produce absorption of the entire bone-matrix or condensation of its cells. Certainly, by making continued pressure upon the teeth in this direction, you do not produce displacement sufficient to accomplish what you evidently have done. Have you any explanation as to what change takes place in the bones structurally or anatomically?

Dr. Case: It is certainly very gratifying to me to hear so much said in praise of that which I have been able to accomplish, and it is especially pleasant to receive in this public manner the indorsement of my efforts, and the verification of the truthfulness of these models which I have presented for your inspection, from men who are well known in the profession, and who have been intimately connected with a number of the cases I have treated.

When I asked your committee to invite Drs. Cushing and Reid to open the discussion of my paper, it was because I learned they were to be here to attend your convention, and because they were eminently qualified, as eye-witnesses, to speak of the practicability of the treatment I have employed in a number of cases.

The suggestions made by Dr. Hoff, in relation to the exact movement that really takes place, is something that it is impossible for me to answer. In the proceedings of the World's Columbian Congress, I mentioned the relation of the intermaxillary bone and its development, and, without giving any special opinion myself, I said that it was possible that that had something to do with the ease with which the four anterior teeth could be moved forward and, possibly, back. The subject of the movement of the roots of the teeth, even in a lateral direction, is one upon which there has been, for years, a great difference of belief among leading specialists in this department of dentistry; while the forward or backward movement of the roots of the anterior teeth with the entire bone in which they are imbedded is a subject so new and of such a radical advancement over former accomplishments in this line, it isn't strange that the profession are surprised and slow to accept or practice it.

[*To be Continued.*]

